# Supplementary material for: N,Nʹ-Diarylurea Derivatives (CTPPU) Inhibited NSCLC Cell Growth and Induced Cell Cycle Arrest through Akt/GSK-3β/c-Myc Signaling Pathway
Source: Int J Mol Sci. 2023 Jan 10;24(2):1357. doi: 10.3390/ijms24021357 (PMC9866857; doi:10.3390/ijms24021357)

Supplementary information

Table S1. Apoptotic cells (%) determined by Hoechst33342/PI staining in three NSCLC cell lines after treatment with CTPPU or CT-(4-OH)-PU for 24 h.

	**CTPPU**		**CT-(4-OH)-PU**	
**Cell line**	**Concentration**	**Apoptotic cells (%)**	**Concentration**	**Apoptotic cells (%)**
H460	control	1.03 ± 0.21	control	0.91 ± 0.12
	25 µM	3.00 ± 0.49	25 µM	1.22 ± 0.50
	50 µM	4.06 ± 0.64	50 µM	1.28 ± 0.08
	100 µM	8.78 ± 1.52	100 µM	7.39 ± 0.64
A549	control	0.72 ± 0.14	control	0.72 ± 0.14
	25 µM	8.19 ± 0.98	25 µM	0.95 ± 0.24
	50 µM	11.12 ± 1.17	50 µM	3.41 ± 0.99
	100 µM	14.18 ± 1.04	100 µM	9.75 ± 1.23
H292	control	0.61 ± 0.12	control	0.65 ± 0.03
	25 µM	1.06 ± 0.07	25 µM	1.26 ± 0.31
	50 µM	1.95 ± 0.78	50 µM	1.66 ± 0.49
	100 µM	6.27 ± 1.92	100 µM	4.33 ± 1.87

Note: values (mean ± Std. Deviation), n = 3

Table S2. Percentage of apoptotic cells determined by flow cytometry in three NSCLC cell lines after 24 h of treatment with CTPPU.

**Cell line**	**CTPPU (µM)**	**Early apoptosis (%) (Annexin V+/PI-)**	**Late apoptosis (%) (Annexin V+/PI+)**	**% Apoptotic cells (Annexin V+)**
H460	0	2.76 ± 0.56	3.22 ± 1.02	5.98 ± 0.54
	25	4.47 ± 0.63	1.89 ± 0.38	6.35 ± 0.98
	50	4.17 ± 1.46	1.90 ± 0.53	6.07± 1.98
	100	5.88 ± 0.63	3.04 ± 0.42	8.91 ± 0.96
A549	0	3.19 ± 0.36	4.41 ± 0.54	7.60 ± 0.90
	25	6.23 ± 0.72	3.34 ± 1.43	9.57 ± 1.63
	50	8.49 ± 1.15	2.64 ± 0.85	11.12 ± 1.67
	100	10.94 ± 1.68	3.59 ± 1.21	14.54 + 2.31
H292	0	0.98 ± 0.15	1.94 ± 0.13	2.92 ± 0.22
	25	0.90 ± 0.32	1.69 ± 0.10	2.59 ± 0.40
	50	0.91 ± 0.34	1.70 ± 0.14	2.61 ± 0.48
	100	2.20 ± 1.17	2.75 ± 0.22	4.94 ± 1.11

Note: values (mean ± Std. Deviation), n = 3

Table S3. % Cell viability determined by MTT assay in three NSCLC cell lines after 24 h of treatment with CTPPU or CT-(4-OH)-PU at a concentration of 100 µM.

**NSCLC cell line**	**% Cell viability**	
	**CTPPU**	**CT-(4-OH)-PU**
H460	50.61 ± 7.84	66.42 ± 3.20
A549	48.30 ± 2.45	79.81 ± 10.5
H292	58.94 ± 6.35	89.17 ± 1.77

Note: values (mean ± Std. Deviation), n = 3

Table S4. Cell cycle distribution determined by flow cytometry in two NSCLC cell lines after 24 h of treatment with CTPPU.

**H460**		**H292**	
**SubG1**	**Cell number (%)**	**SubG1**	**Cell number (%)**
Control	0.23 ± 0.11	Control	0.15 ± 0.04
25 µM	0.17 ± 0.08	25 µM	0.37 ± 0.18
50 µM	0.26 ± 0.08	50 µM	0.40 ± 0.12
**G0/G1**		**G0/G1**	
Control	43.85 ± 0.02	Control	37.66 ± 1.76
25 µM	53.98 ± 1.22	25 µM	44.20 ± 0.34
50 µM	53.58 ± 0.46	50 µM	45.97 ± 0.87
**S**		**S**	
Control	15.81 ± 0.35	Control	20.08 ± 2.07
25 µM	19.50 ± 0.51	25 µM	21.78 ± 0.49
50 µM	18.27 ± 0.38	50 µM	19.92 ± 0.74
**G2/M**		**G2/M**	
Control	40.12 ± 0.42	Control	42.11 ± 2.68
25 µM	26.36 ± 0.78	25 µM	33.55 ± 0.79
50 µM	27.90 ± 0.76	50 µM	33.71 ± 1.06

Note: values (mean ± Std. Deviation), n = 3

Figure S1. The original images of Western blot in Figure 5A.

Figure 5A (left)

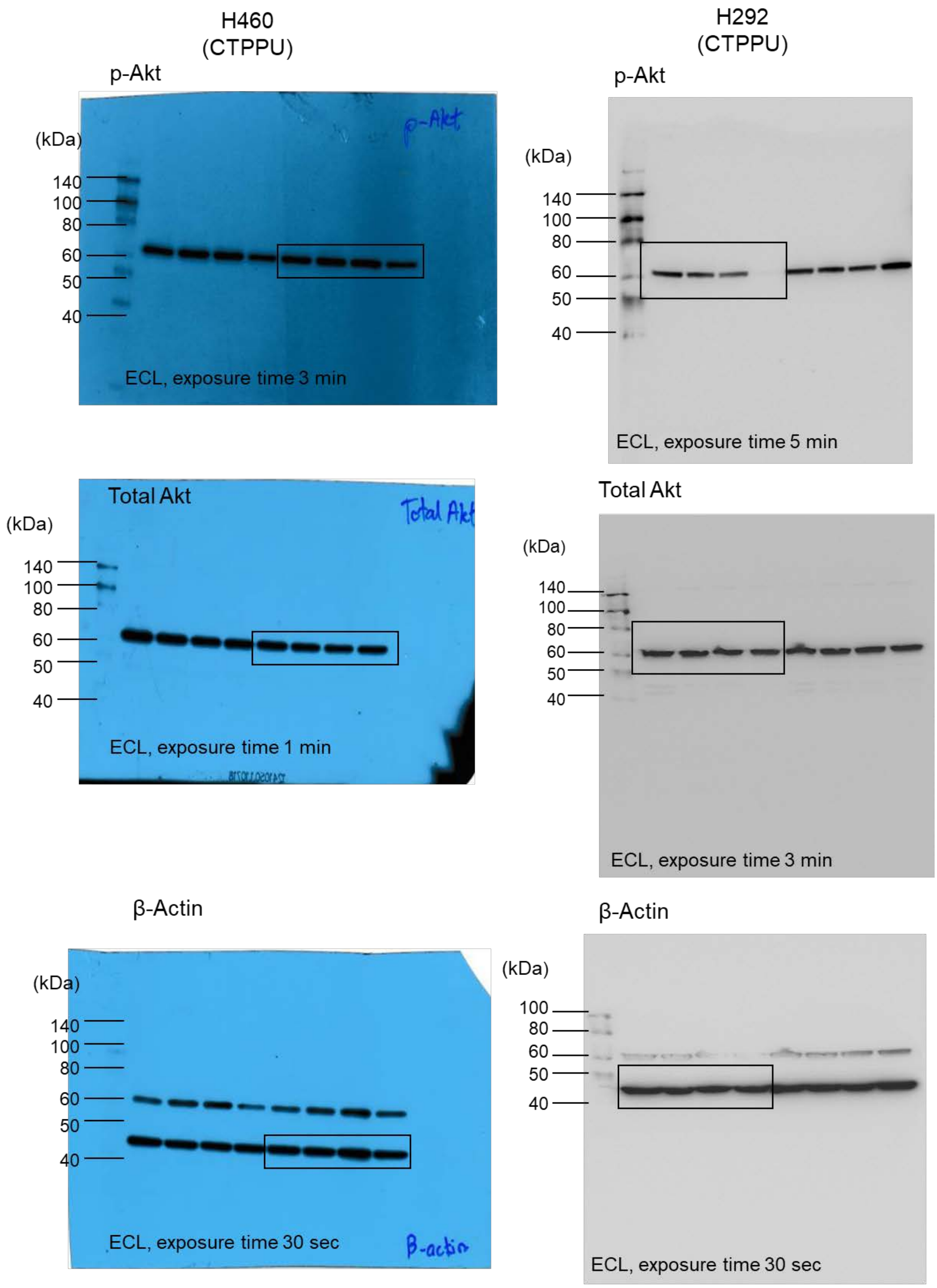

Figure 5A (right)

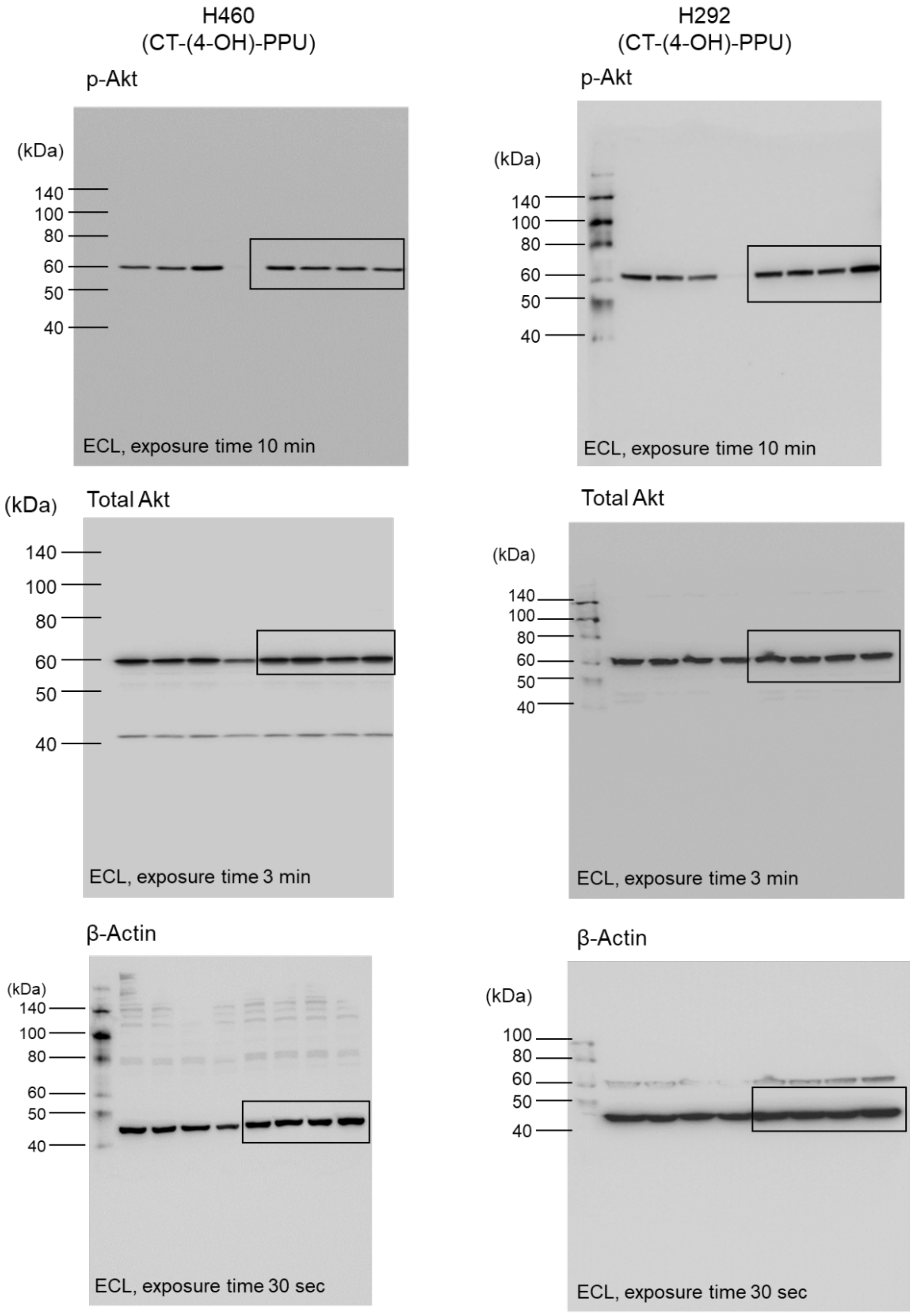

Figure S2. The original images of Western blot in Figure 5C.

<u>Figure 5C (left)</u>

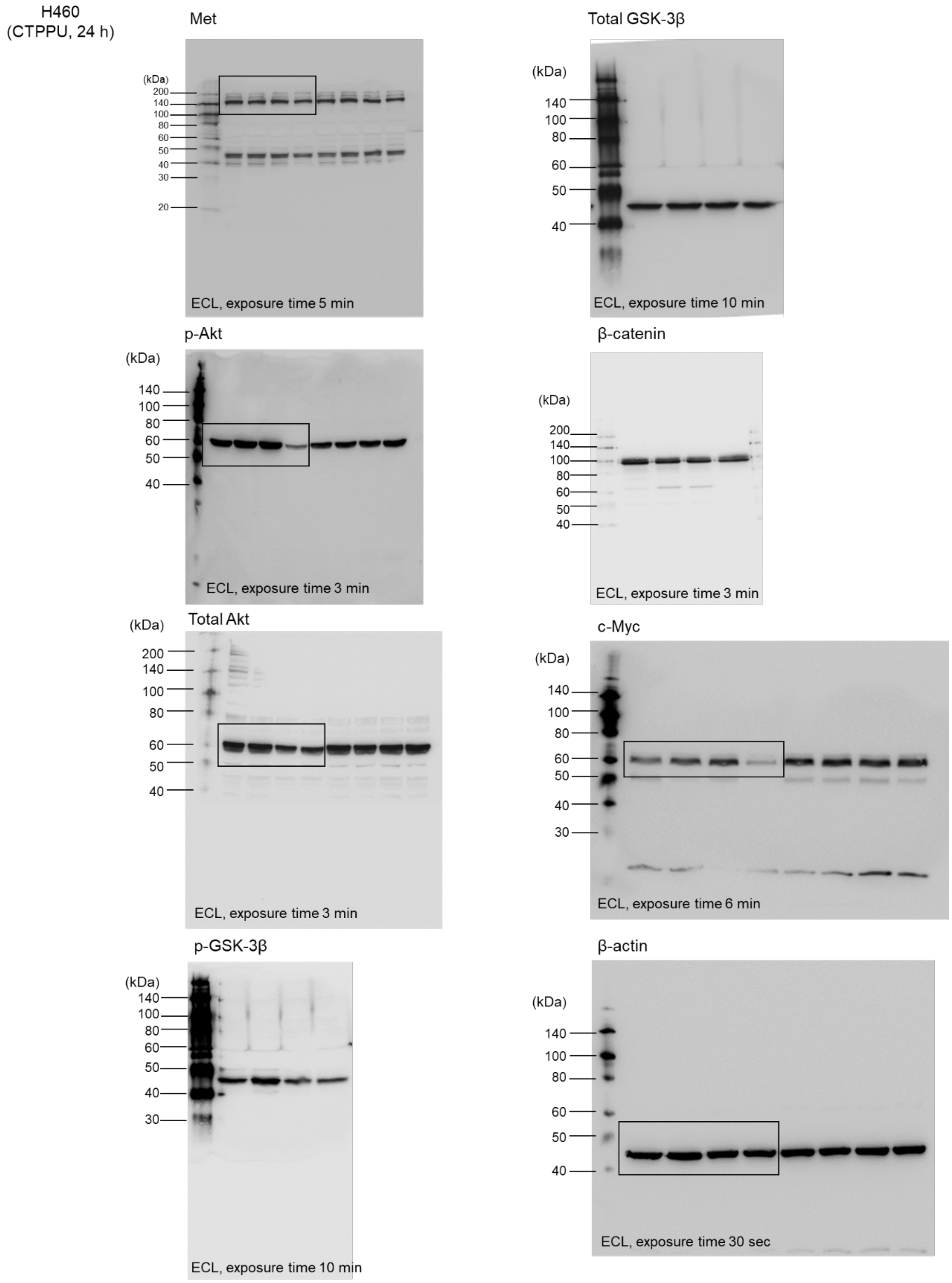

<u>Figure 5C (right)</u>

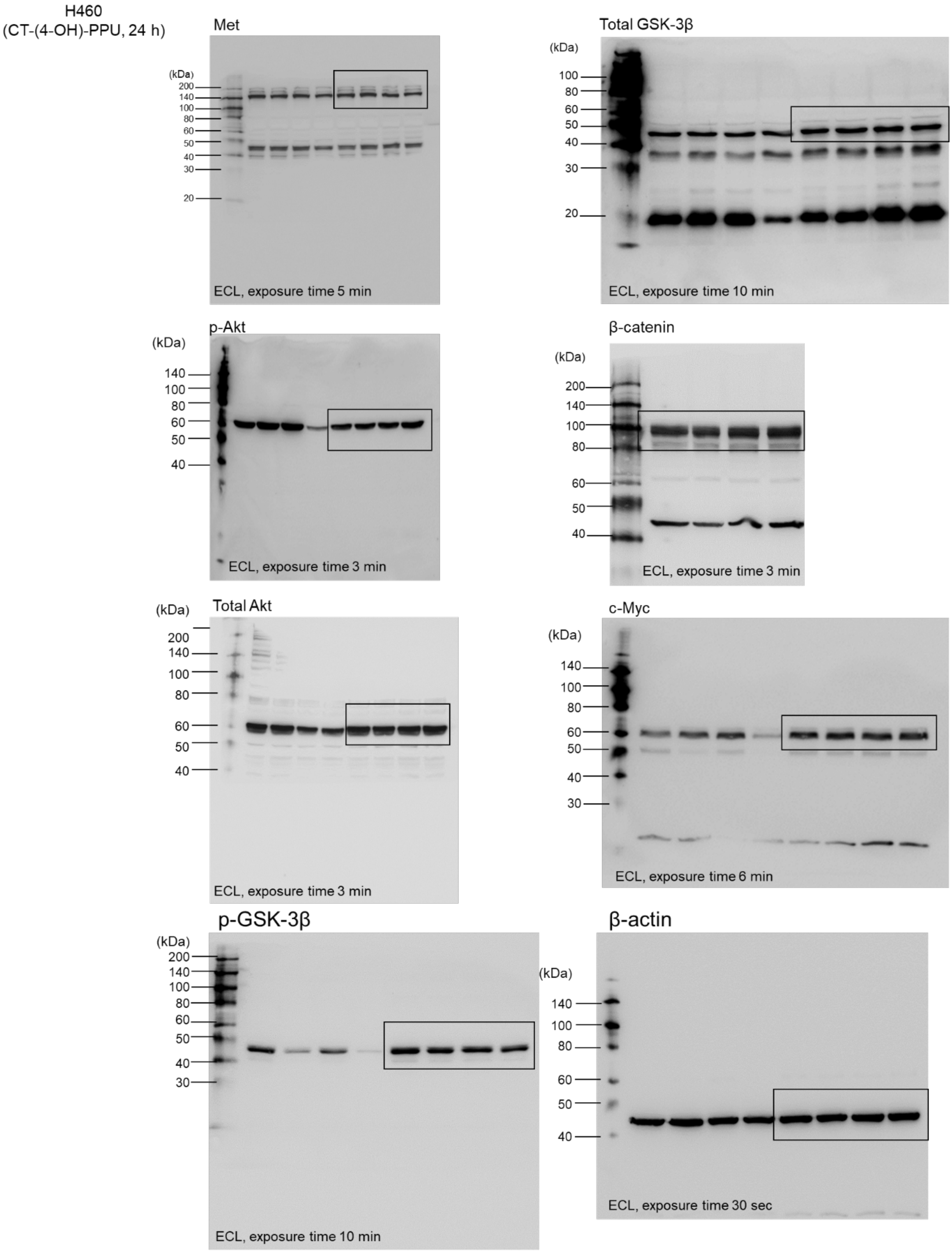

Figure S3. The induction of senescence in H460 cells in response to CTPPU.

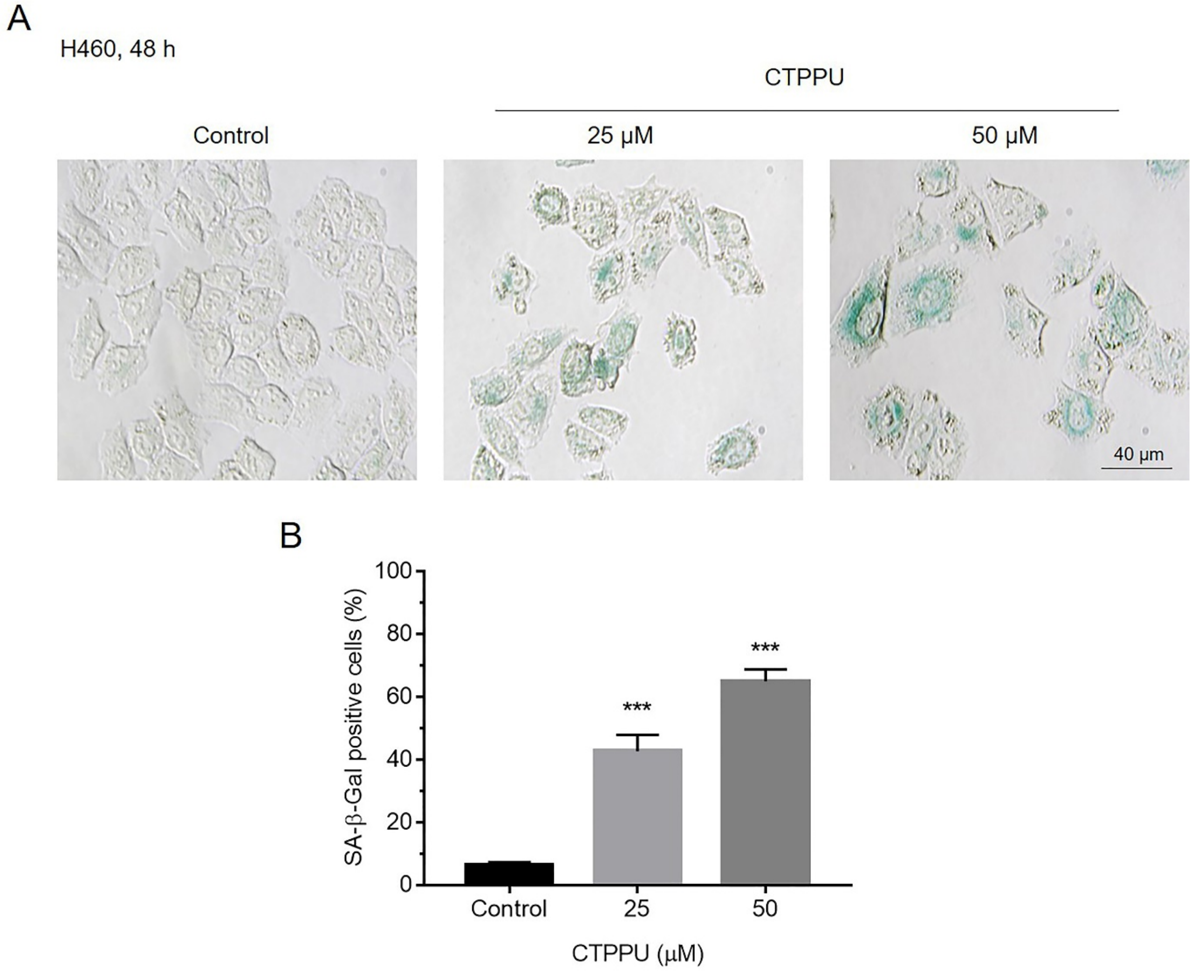

Supplementary Figure S3. (A) H460 cells were treated with various concentrations (0, 25, and 50 μM) of CTPPU for 48 h. Cell morphological changes were observed under a microscope (×400 magnification), and cells were subjected to SA-β-gal staining (B) The percentages of SA-β-gal positive cells were counted in five random areas. The results are presented as mean ± SD. Error bars are standard deviations. Significant differences are indicated as ***$p < 0.001$ compared with the control.

Figure S4. The original images of Western blot in Figure 9J

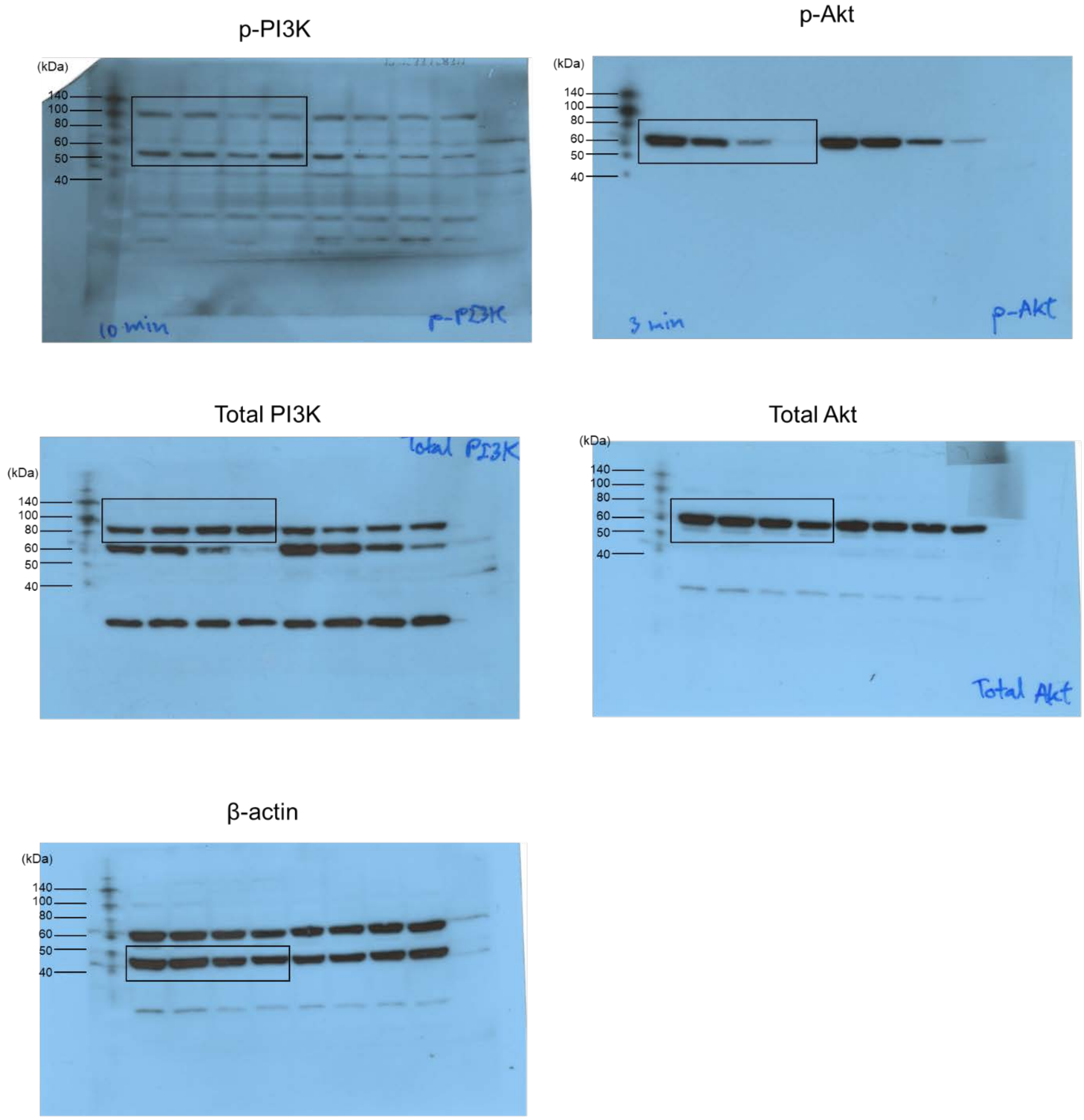

Supplement: Supplementary file 1 [file ijms-24-01357-s001.zip › ijms-2154907-supplementary.pdf]
